# Robot’s Social Gaze Affects Conflict Resolution but not Conflict Adaptations

**DOI:** 10.5334/joc.189

**Published:** 2022-01-06

**Authors:** Francesca Ciardo, Agnieszka Wykowska

**Affiliations:** 1 Istituto Italiano di Tecnologia, Via Enrico Melen 83, 16152 Genova, IT

**Keywords:** Conflict resolution, Conflict adaptations, Social Gaze, Human-Robot Interaction

## Abstract

Robots are a new category of social agents that, thanks to their embodiment, can be used to train and support cognitive skills such as cognitive control. Several studies showed that cognitive control mechanisms are sensitive to affective states induced by humor, mood, and symbolic feedback such as monetary rewards. In the present study, we investigated whether the social gaze of a humanoid robot can affect cognitive control mechanisms. To this end, in two experiments, we evaluated both the conflict resolution and trial-by-trial adaptations during an auditory Simon task, as a function of the type of feedback participants received in the previous trial from the iCub robot, namely, mutual or avoiding gaze behaviour. Across three experiments, we compared the effect of mutual, avoiding (Exp1 and Exp2), and neutral (Exp3) gaze feedback between screen-based (Exp1) and physically embodied setups (Exp2 and Exp3). Results showed that iCub’s social gaze feedback modulated conflict resolution, but not conflict adaptations. Specifically, the Simon effect was increased following mutual gaze feedback from iCub. Moreover, the modulatory effect was observed for the embodied setup in which the robot could engage or avoid eye contact in real-time (Exp2) but not for the screen-based setting (Exp1). Our findings showed for the first time that social feedback in Human-Robot Interaction, such as social gaze, can be used to modulate cognitive control. The results highlight the advantage of using robots to evaluate and train complex cognitive skills in both healthy and clinical populations.

## Introduction

Complex cognitive skills in humans are the result of interaction with the environment, including social interactions. Indeed as stated by Humpreys, *“[…] cognitive functions have evolved to their high level because they have been driven by the complexities of social livin*g” (Humphreys, 1976, p. 307). Nowadays, social interactions are not only limited to other humans but also include artificial agents, such as robots. The pivotal role that robots can play in promoting and supporting human cognitive skills is demonstrated by the increased interest in developing socially assistive robotics in the last years (Chevalier et al., 2019; Wykowska, 2020). Indeed, as social agents, robots represent a perfect combination of symbolic (e.g. language and communicative gestures) and artifactual instruments that, according to the socio-cultural approach (e.g., Vygotskij, 1932), are necessary to ensure and support the development of cognitive skills such as communication and behaviour regulation (Ciardo & Wykowska, 2020).

Among cognitive skills, one of the most crucial is cognitive control, which is the ability to adapt our behaviour to maintain and achieve task goals by reducing the cognitive conflict in task execution, when simultaneous and mutually incompatible goal representations competing for a single response are activated. Several studies showed that cognitive conflict can be modulated by either enhancement of processing of task-relevant information (e.g., Egner & Hirsh, 2005) or by inhibition of task-irrelevant features (e.g., Braver, 2012; Ridderinkhof, 2002). The former refers to the adjustments of task parameters and priority settings occurring at the level of task strategy (e.g. Braver, 2012; Logan, 1985), resulting in a better **conflict resolution** within a given trial. Namely, resources are allocated to prevent the negative impact of a cognitively demanding event on task performance (e.g., Marini, Chelazzi, & Maravita, 2013). This form of control differs from online **conflict adaptations** in performance triggered by the conflict experienced in the previous trial (e.g., Botvinick, Braver, Barch, Carter, & Cohen, 2001; Braver, 2012).

The mechanisms allowing humans to deal with cognitive conflict have been studied in the literature using tasks that manipulate conflict, such as the Flanker task (Eriksen & Eriksen, 1974), the Stroop task (Stroop, 1992), the task-switching paradigms (e.g. Braem et al., 2012) or the Simon task (Simon & Rudell, 1967; for a review see Proctor & Vu, 2006). In a Simon task, participants are asked to respond to a feature of a target stimulus usually presented visually on the screen. The feature, colour, for example, determines which button a participant is supposed to press (e.g., left button for red targets and right button for green targets). Most importantly, the target stimuli are presented laterally on the screen, and the responses are also lateral. This means that each target will require a spatially corresponding or non-corresponding response (in the example above, if the red target is presented on the left, the response will be spatially corresponding, but if the red target is presented on the right, the response will not be corresponding). Typically, corresponding target-response mappings elicit faster responses (and/or lower error rates) than non-corresponding mapping (e.g. Simon & Rudell, 1967), although the spatial configuration is completely irrelevant to the task. The correspondence effect in a Simon task is termed the Simon effect (SE), and it has been explained as resulting from a conflict during response selection (e.g., Rubichi & Pellicano, 2004) between two alternative response codes, one generated based on task instructions and the other automatically activated through pre-existing associations linking a stimulus to its spatially corresponding response (e.g., De Jong, Liang, & Lauber. 1994). The automatic association between stimulus and response codes has laid the grounds for the Theory of Event Coding (Hommel et al., 2001) which postulates a common code between perception and action. Most importantly, due to the automatic activation of a stimulus-response code that incorporates spatial information, even though it is task-irrelevant, the response selection process is facilitated in corresponding trials, in which the two activated response codes overlap, leading to faster reaction times (RTs). Conversely, conflict occurs in non-corresponding trials in which the irrelevant and the relevant stimulus dimensions activate different response codes, thus impeding RTs. Interestingly, SE is reduced, null, or even reversed for trials that follow a non-corresponding trial, while it is consistently observed for trials following a corresponding trial (e.g., Ciardo, Ricciardelli, & Iani, 2019; Hommel, Proctor, & Vu, 2004). These trial-by-trial adaptations have been taken as evidence that the conflict experienced in a given trial is accompanied by changes aimed at preventing the recurrence of the conflict in the next trial (for a review, see Mansouri, Tanaka, & Buckley, 2009; but see also Hommel et al., 2004 for an alternative account).

Although the independence of **conflict resolution** and **conflict adaptation** mechanisms is still debated (see Egner, Ely, & Grinband, 2010; Scherbaum, Fischer, Dshemuchadse, & Goschke, 2011 for an alternative account), they show different developmental strategies and are differently affected by individual and contextual factors. Indeed, evidence shows that individual differences such as age, cognitive style, or psychiatric disorders (e.g. eating or mood disorders) are reflected in conflict resolution, but not in conflict adaptations (Larson, Clawson, Clayson, & South, 2012; Iani, Stella, & Rubichi, 2014; Iani, Ciardo, Ricciardelli, & Nicoletti, 2016; Bartholdy, et al., 2017).

Another factor to which cognitive control seems to be sensitive is the affective state^1^ induced by reward, humor, and mood (see van Steenbergen, 2015 for a review). For instance, Kanske and Kotz investigated, in a series of studies, whether and how conflict resolution is modulated by the emotional content of the stimuli. Using different types of conflict tasks, the authors showed that both positive and negative words can speed up conflict resolution (i.e. reduced Flanker and Simon effects for negative and positive targets, respectively, Kanske & Kotz, 2010; 2011;2012). A similar result was reported by Yamaguchi & Nishimura (2018), who manipulated monetary reward. Specifically, using a Flanker task, the authors showed that conflict was reduced (i.e. smaller Flanker effect) for contingent-reward trials compared to trials in which the reward was randomly assigned (Exp2, Yamaguchi & Nishimura, 2018). However, this was not the case for conflict adaptations, as the authors showed that both contingent and non-contingent rewards had little effect on trial-by-trial effects, highlighting the dissociation between conflict resolution and conflict adaptation.

Online conflict adaptations have also been shown to be modulated by affective states. Padmala and colleagues (2011) presented both neutral and high-arousing negative images (i.e., mutilated bodies) in-between Stroop task trials. The results showed that following negative pictures the usual trial-by-trial adaptations did not occur. Similarly, van Steenbergen and colleagues (2010) investigated the effect of positive and negative mood-induction before performing a conflict-evoking Flanker task. Results showed that trial-by-trial adaptations were affected by the pleasure of the induced mood, with a larger reduction in cognitive conflict (i.e. less interference) for subjects assigned to the negative mood condition (i.e., anxious and sad) compared to those assigned to the positive mood group (i.e., calm and happy). Similar results have been also reported from studies that manipulated reward, showing an enhanced cognitive control (i.e. less interference) following negative feedback (i.e. losses or small gains) than following gain feedback (e.g., Braem et al., 2012; Stürmer et al., 2011; Schuch, Zweerings, & Koch, 2017; but see also Yamaguchi et al. 2020 for a series of studies failing in replicating the effect of affective states on trial-by-trial effects).

It has been proposed that affective states (mood, reward, or emotions) seem to enhance cognitive control in an affect-congruent manner (cf. Cabanac, 1992; van Steenberg, 2014). That is, negative affective states might influence cognitive conflict for the demanding cases, i.e. incongruent (conflict) trials, as those also trigger a negative and aversive state (Botvinick, 2007). A recent systematic review by Dignath and colleagues (2020) underlies how the effect of affective states on conflict adaptations is influenced by the way the affective state is induced during the task. While tonic affective states, like mood, showed consistent results in support to the affect-congruent hypothesis, studies that manipulated affective states in a transient way, such as monetary reward on random trials, showed mixed and contradictory results, mostly due to the heterogeneity of the tasks and manipulations across studies (Dignath et al., 2020).

The studies reviewed so far manipulated mainly emotions and monetary rewards, focusing more on the distinction between affective states and motivational factors. However, in everyday life during social interactions, we are exposed to several non-verbal social communication signals, such as facial expression, body language, and social gaze, that can induce affective states or, as feedback, modulate our motivation. For instance, when we are taking an exam, a smiling examiner looking towards us can induce a more relaxed state and, thereby, help in focusing on our task.

The social gaze is defined as the use of gaze direction with communicative intent (e.g., Emery, 2000). Two social gaze behaviours that induce affective states are: avoiding and mutual gaze. Converging evidence suggests that real-time mutual gaze increases arousal and evokes a positive affective state (but see also Jarick & Kingstone, 2015, for evidence on mutual gaze being perceived as socially uncomfortable and aversive). For instance, mutual gaze has been associated with increased skin conductance, heart rate, suppression of alpha activity, and increased engagement compared to avoiding gaze (e.g., Hietanen et al., 2008; Pönkänen, Peltola, & Hietanen, 2011; Kompatsiari et al., 2021a). Interestingly, such a modulation appears to be null or reversed when the social gaze is presented within screen-based setups (Hietanen et al., 2008; Pönkänen, Peltola, & Hietanen, 2011).

In a recent series of studies, Kompatsiari and colleagues showed that similar effects are elicited also in interaction with a robot (e.g., Komptsiari et al., 2018; 2019; 2021a). Specifically, when the humanoid robot iCub (Metta et al., 2010) established real-time mutual gaze it was judged as more engaging and human-like compared to when it avoided eye contact. Similarly, Schellen et al. reported that mutual or avoiding gaze presented after participants’ choice in a decision-making task induced a change of strategy in the subsequent trial. The decision-making task was related to giving honest or deceptive feedback to iCub. The authors found that fewer deception choices occurred after trials in which iCub established mutual gaze as a feedback (Schellen, Bossi, & Wykowska, 2021). In a recent EEG study, Kompatsiari and colleagues examined oscillatory brain response to mutual and averted gaze established by the iCub robot during an attentional cuing task (Kompatsiari, Bossi, & Wykowska, 2021). Results showed that mutual and avoiding gaze differently affected the desynchronization of alpha-band activity, suggesting that following mutual gaze, participants were inhibiting task-irrelevant information to a lesser extent, relative to averted gaze. Such a result is in line with the hypothesis that cognitive control is reduced in social situations, as the need of monitoring another agent reduces the attentional resources allocated to the task (e.g. Huguet et al., 2014). In line with this latter hypothesis, there is evidence showing that conflict effects, such as the Stroop effect, are reduced in the presence of another human or a humanoid agent (Huguet et al.,1999; Spatola et al., 2018; see Belletier et al 2019 for a review).

To summarize, the social gaze is a powerful (often implicit) feedback that in social interactions can induce affective states. The effectiveness of mutual or averted gaze seems to be stronger for real-time (embodied) interactions compared to screen-based setups (Hietanen et al., 2008; Pönkänen, Peltola, & Hietanen, 2011), and it has been widely replicated in Human-Robot Interaction (HRI) (Kompatsiari et al., 2018; 2019; 2021a). The fact that robotic agents can exert similar effects on cognitive mechanisms to those induced by other humans is recent but consistent evidence across several tasks. For instance, it has been shown that human and non-anthropomorphic robotic agents similarly affect sense of agency (e.g. Ciardo et al., 2020), motor preparation, and attention allocation both at the behavioural and neural levels (Hinz et al., 2021; Komptsiari et al. 2018).

The increasing interest in developing socially assistive robotics for training purposes (Ciardo & Wykowska, 2020; Wykowska, 2020) calls for the need to study whether the social signals of a robot can be used as feedback to modulate cognitive control mechanisms. Therefore, the present study, aimed to i) investigate whether using the social gaze of a robot as feedback modulates cognitive control; ii) understand if the social gaze feedback of a robot modulates cognitive control by conflict resolution mechanism (i.e., adjusting task parameters and priority settings before the occurrence of cognitively demanding events), or by affecting also trial-by-trial adaptations; iii) test the impact of physical embodiment. In three experiments, we evaluated both the SE overall and trial-by-trial adaptations as a function of the type of social gaze feedback that iCub exhibited at the end of the previous trial, namely mutual, avoiding, or neutral gaze.

We implemented an auditory Simon task (Simon & Rudell, 1967) to avoid having too much visual information to process while performing the task and to make sure that visual attention was focused on iCub’s face. In Exp1 and Exp2, we compared the effect of the social gaze feedback between screen-based setups and setups involving physically present embodied iCub, respectively. In Exp 3, we ran a follow-up control in which the robot was not providing any feedback to the participants. This was done to estimate the baseline magnitude of the SE elicited by our embodied setup.

Based on the reviewed literature (Hietanen et al., 2008; Kompatsiari et al., 2018; 2019), we hypothesized that iCub’s mutual and avoiding gaze should influence cognitive control mechanisms by inducing affective states. According to the hypothesis that affective states modulate cognitive control in an affect-congruent way (van Steenberg, 2014), and the existing evidence showing that mutual gaze induces positive affective states (Hietanen et al., 2008; Pönkänen, Peltola, & Hietanen, 2011; Kompatsiari et al., 2021a), the SE was expected to be reduced following avoiding gaze feedback or increased after mutual gaze feedback. Specifically, if the social gaze interacts with conflict resolution, then its modulatory effect should be evident at the task strategy level, resulting in a smaller SE within a given trial. On the other hand, if the social gaze modulates online conflict adaptations, then different trial-by-trial effects should emerge across the two gaze conditions. Concerning the comparisons between screen-based and embodied setups, we hypothesized that the social gaze (mutual or avoiding) should be more effective when manipulated in physical presence and in real-time compared to when it is depicted on a screen. Thus, the modulating effect of social gaze should be stronger for embodied setup (Exp2) compared to the screen-based setup (Exp1).

## Experiment 1

### Participants

The sample size was estimated via a priori power analysis using G*Power. The analysis yielded a sufficient number of 15 participants for the within-subject design [dz = 0.40, α = 0.05, and 1-β = 0.80].Twenty-one participants (5 males; mean age: 24.4 ± 3.5 years) took part in the study. All participants had normal or corrected-to-normal vision and were not informed about the purpose of the experiment. All participants gave their informed written consent. All experiments were conducted under the ethical standards laid down in the 1964 Declaration of Helsinki and were approved by the Local Ethical Committee (Comitato Etico Regione Liguria). The data of one participant have been excluded due to a technical failure of the program. Therefore, data of twenty participants were further analyzed.

### Materials and Methods

#### Apparatus and Stimuli

The experiment was carried out in a dimly lit and noiseless room. The participant was seated facing a 22” LCD monitor driven by a 2.90 GHz processor computer at a viewing distance of 60 cm. Stimuli were ‘high’ (800 Hz) or ‘low’ (400 Hz) tones presented through Sennheiser HD 569 headphones (10–28000 Hz). Responses were executed by pressing the ‘q’ or the ‘p’ keys on the QWERTY keyboard with the left or the right index finger, respectively. Two videos showed the iCub robot establishing real-time mutual or avoiding gaze (from a participant’s perspective). The keyboard was located centrally with respect to the body midline. Stimulus presentation, response timing, and data collection were controlled by Psychopy software (v.2020.1.3).

#### Procedure

Participants were instructed to respond as quickly and as accurately as possible to the tone pitch presented to one of their ears through either left or right headphones. Participants were asked to ignore the tone’s spatial location. Half of the participants responded to the high tone by pressing the “q” key and to the low tone by pressing the “p” key, the other half experienced the opposite stimulus-response mapping. Each trial began with the presentation of a white fixation cross in the center of a black screen for 900 ms, then the fixation cross turned to yellow for 1100 ms. 200 ms after the fixation cross changed colour the tone was played, see D’ascenzo et al. (2018) for a similar procedure. Given that evidence showed that left and right auditory stimuli produce a decreasing Simon effect distribution (Xiong & Proctor, 2015) due to the dissipation of the automatic activation of the spatial corresponding response, the maximum time allowed to respond was 1000 ms. After a response was made, or the maximum time allowed expired, a video depicting iCub establishing Mutual or Avoiding gaze was presented for 6000 ms (see ***Video 1***). The inter-trial interval was 1000 ms. Participants were also instructed to pay attention to the iCub following their response because at the end of the experiment they would be asked to answer questions about its behaviour. At the end of each block, feedback indicating the cumulative amount of correct responses, and the average response time across the experiment was presented.

**Video 1:** Recording of trial procedure in Experiment 1.

The task consisted of 4 blocks with 64 trials each. A short practice of 8 trials preceded the task. In each block, the trial sequence was controlled so that each trial was preceded by either a corresponding (C) or non-corresponding (NC) trial, with equal probabilities to be preceded by Avoiding or Mutual Gaze condition. As a result, in both Avoiding and Mutual gaze conditions, four different trial sequences occurred (*C*–C, *C*–NC, *NC*–C, *NC*– NC, with italics denoting trial *n–1*).

### Data Analysis

We excluded from analyses the first trial of each block, trials that were preceded by an incorrect response, incorrect responses, and correct responses faster than 150 ms and slower than 1000 ms (7.0%of the administered trials). Mean correct RTs were submitted to a repeated-measures analysis of variance (ANOVA) with Trial *n–1* Correspondence (*C* vs. *NC*), Trial n Correspondence (C vs. NC), and Preceding Gaze Feedback (Avoiding vs. Mutual) as within-subject factors. When necessary, comparisons were performed using paired samples t-tests. Analysis was run using the Jasp software (v.0.9.2).

## Results and Discussion

The analysis revealed a significant main effect of Trial n Correspondence, F(1, 19) = 156.74, p < .001, η_p_^2^ = .89, indicating faster responses for corresponding (M = 464 ms, SE = 2.1 ms) than for non-corresponding trials (M = 515 ms, SE = 2.2 ms), indicating the classical SE. The correspondence effect was modulated by correspondence sequence, as indicated by the significant interaction between Trial n Correspondence and Trial *n–1* Correspondence, F(1, 19) = 14.18, p < .001, η_p_^2^ = .43. Planned comparisons showed a 59-ms SE after a corresponding trial, t(19) = 13.3, p < .001, d = 3.0; and a SE of 28-ms after a non-corresponding trial, t(19) = 8.9, p < .001, d = 2.0. Paired simple t-test showed that the two effects differed in magnitude, t(19) = 3.8, p < .001, d = .8. No other main effect or interaction were significant, all ps > .094, see ***Table 1***.

**Table 1:** Mean correct reaction times and standard deviation (in milliseconds) as a function of Experiment (Exp1 vs Exp2), Preceding Gaze Feedback (Avoiding vs Mutual), Trial n – 1 (corresponding, C vs non-corresponding, NC), and Trial n (corresponding, C vs non-corresponding, NC).


	PRECEDING GAZE FEEDBACK	TRIAL N-1	TRIAL N	MEAN	SD

Exp.1	Avoiding	C	C	450	89

			NC	509	106

		NC	C	471	96

			NC	515	98

	Mutual	C	C	461	94

			NC	520	96

		NC	C	474	95

			NC	516	103

Exp.2	Avoiding	C	C	429	99

			NC	481	80

		NC	C	447	91

			NC	465	79

	Mutual	C	C	432	95

			NC	497	95

		NC	C	458	109

			NC	495	95


The results of Experiment 1 showed that the SE was modulated by the correspondence of the preceding trial. Specifically, in line with previous studies, the typical trial-by-trial adaptations occurred (e.g., Ciardo et al., 2019; Iani et al., 2014) with a larger SE evident following a corresponding (59 ms) than a non-corresponding trial (28 ms). These results support the idea that the detection of conflict in trial *n–1* triggers adaptations that are aimed at eliminating the impact of spatial S–R correspondence on response selection in the following trial (Braver, 2012; Ridderinkhof, 2002). No effect of preceding gaze feedback emerged. Such results may suggest that the mutual and avoiding gaze feedback of a humanoid robot may not act as an affective signal and do not affect performance in the subsequent trial. However, in Experiment 1 mutual and avoiding gaze feedbacks were manipulated through a robot face presented on the screen. Such a setup may lack ecological validity. Indeed, several studies showed that embodiment plays a crucial role when investigating social cognition mechanisms (e.g. Vasco et al., 2019; Wiese, Metta, Wykowska, 2017), especially in the context of mutual gaze, with higher arousal and faster responses associated with real-time mutual gaze compared to direct gaze presented on a screen (Hietanen et al., 2008; Pönkänen et al., 2011). Taking this into account, we designed Experiment 2, in which we implemented the screen-based paradigm to a 3-D physical-presence setup in which the mutual and avoiding gaze feedbacks from iCub were displayed in real-time.

## Experiment 2

### Materials and Methods

#### Participants

Twenty-five new participants (7 males; 3 left-handed; Mean age: 25.6 ± 4.2 years) took part in the study. All participants had normal or corrected-to-normal vision and were not informed about the purpose of the experiment. All gave their informed consent before participating and the study was conducted under the same ethical procedures and protocol as in Experiment 1. The data of five participants were excluded due to: technical failure of the robot (2), technical failure of the program (2), or because the participant was unable to perform the task (1). Therefore, data of twenty participants were further analyzed.

#### Apparatus and Stimuli

**Figure 1:**
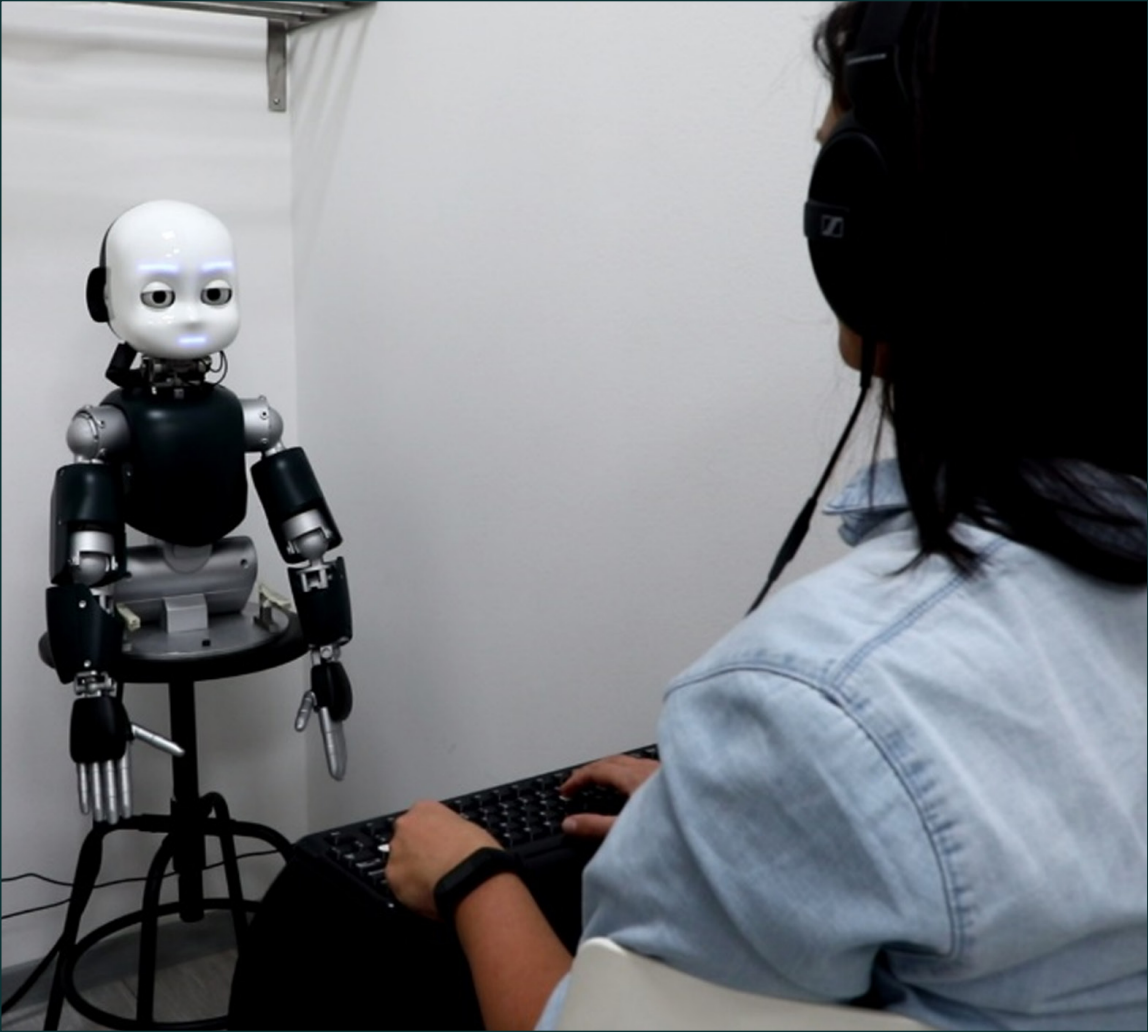
Experimental setup in Experiment 2.

The experiment was carried out in a dimly lit and noiseless room. The participant was seated facing the iCub robot at a viewing distance of 70–80 cm (see ***Figure 1***). We used a version of the iCub robot consisting of a full robotic head and a 3D printed torso mounted on a stool. The iCub’s eyes have 3 degrees of freedom (common tilt, vergence, and version) and three additional degrees of freedom in the neck (roll, pitch, yaw). To control the eyes and the neck of iCub, we used the YARP (Yet Another Robot Platform, Metta, Fitzpatrick, & Natale, 2006) Python wrappers.

In our procedure, to make iCub gaze at a specific location, a 6-DOF gaze controller has been used (Roncone, Pattacini, Metta, & Natale, 2016). This controller uses inverse kinematics to find eyes and neck positions to make the robot look at certain 3D Cartesian coordinates. In our experiment, the target location was predefined for the Avoiding gaze feedback (x: 0°,y: –26°, z: 3°), whereas, in the Mutual gaze feedback coordinates of target location were calculated online in each trial, to make the robot establish eye contact in real-time (see ***Video 2***). Participant’s eyes were detected using a face detection algorithm applied to the images coming from the camera sensors placed in the eye bulbs of the iCub robot (Cao, Hidalgo Martinez, Simon, Wei, Sheikh, 2017). The vergence of the eyes was set to 3 degrees and maintained constant. The trajectory time for the movement of eyes and neck was set to 500 ms, to maintain the impression of a smooth and naturalistic movement.

**Video 2:** Recording of trial procedure in Experiment 2.

Stimuli were the same as those used in Experiment 1. Responses were executed as in Experiment 1. The keyboard was placed on the participant’s lap centrally with respect to the body midline. Stimulus presentation, response timing, and data collection were controlled as in Experiment 1.

#### Procedure

As in Experiment 1, participants were instructed to discriminate the tone pitch in one of the headphones, ignoring its spatial location. Participants were also instructed to pay attention to the iCub because at the end of the experiment they would be asked to answer questions about its behaviour. Each trial started with the robot depicting a neutral expression and closed eyes with the neck located at the rest position. Following 1500 ms the expression of the robot turned from neutral to surprise for 1700 ms. 200 ms after the facial expression changed the stimulus tone was played. The maximum time allowed to respond was set at 1000 ms from stimulus onset. After a response was given, or the allowed response time elapsed, the robot opened the eyes and moved the neck and the eyes toward the Avoiding or Mutual gaze target location, see ***Video 2***. At the end of each block, feedback indicating the cumulative amount of correct responses and the average response time across the task was given to participants. Contrary to Experiment 1, instructions and feedback were given verbally by iCub using the text to speech library: *https://github.com/robotology/speech/tree/master/svox-speech*. The task consisted of 5 blocks of 48 trials each. A short practice of 8 practice trials preceded the task. As in Experiment 1, in each block, the trial sequence was controlled so that each trial was preceded by either a corresponding (C) or non-corresponding (NC) trial, with equal probabilities to be preceded by Avoiding or Mutual Gaze condition. As a result in both Avoiding and Mutual gaze conditions, four different trial sequences occurred (*C*–C, *C*–NC, *NC*–C, *NC*– NC, with italics denoting trial *n–1*).

### Data Analysis

As for Experiment 1, the first trial of each block, trials that were preceded by an incorrect response, incorrect responses, and correct responses faster than 150 ms and slower than 1000 ms were excluded from the analysis (12.0%of the administered trials). Mean correct RTs were submitted to a repeated-measures analysis of variance (ANOVA) with Trial n – 1 Correspondence (C vs. NC), Trial n Correspondence (C vs. NC), and Preceding Gaze Feedback (Avoiding vs. Mutual) as within-subject factors. When necessary, comparisons were performed using paired samples t-tests.

## Results and Discussion

**Figure 2:**
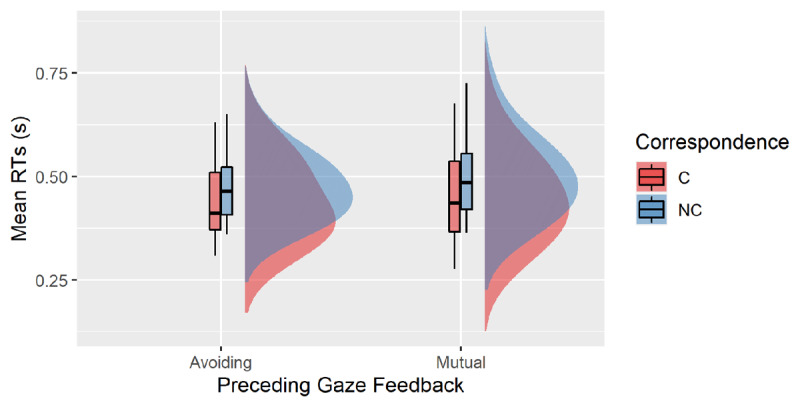
Mean reaction times in Experiment 2 for the corresponding (C) and non-corresponding (NC) Trial n, as a function of Preceding Gaze Feedback (Avoiding vs Mutual).

The analysis on RTs showed that responses were faster following Avoiding gaze (M = 456 ms, SE = 1.9 ms) than Mutual gaze trials (M = 472 ms, SE = 2.2 ms), as indicated by the main effect of Preceding Gaze, F(1, 19) = 69.67, p < .001, η_p_^2^ = .79. The main effect of Trial n Correspondence was significant, F(1, 19) = 69.67, p < .001, η_p_^2^ = .79, with faster responses for corresponding (M = 442 ms, SE = 2.1 ms) than for non-corresponding trials (M = 485 ms, SE = 1.9 ms), indicating a classical SE. This correspondence effect was modulated by correspondence sequence, as indicated by the significant interaction between Trial n Correspondence and Trial *n–1* Correspondence, F(1, 19) = 22.23, p < .001, η_p_^2^ = .54. Planned comparisons showed the typical sequential effects reported in Experiment 1 and in previous studies (e.g., Ciardo et al., 2019; Iani et al., 2014) with a 59-ms SE after corresponding trials, t(19) = 8.8, p < .001, d = 2.0; and a 43 ms SE after a non-corresponding trials, t(19) = 4.9, p < .001, d = 1.1. Paired-samples t-test showed that the two effects differed in magnitude, t(19) = 4.72, p < .001, d = 1.05. Importantly, the two-way interaction between Preceding Gaze Feedback and Trial n Correspondence was significant, F(1, 19) = 6.35, p = .021, η_p_^2^ = .25. Planned comparison showed that a larger SE occurred following Mutual gaze feedback (51 ms) than Avoiding gaze feedback (35 ms), t(19) = 2.59, p = .021, d = .56 (see ***Figure 2***). No other main effects or interactions were significant, all ps > .14.

Results of Experiment 2 showed that mutual and avoiding gaze feedback differently affected the SE in the subsequent trial. Specifically, we found that the SE was smaller in magnitude following avoiding gaze feedback, relative to mutual gaze (35 vs. 51, respectively). Social gaze feedback did not modulate sequential effects, as there was no significant three-way interaction. These results indicate that social gaze feedback modulated conflict within a single trial, but not adaptation across trials. Hence, it seems that the ability to manage conflict within a single trial and the ability to adapt performance based on the conflict experienced in the previous trial depend on different mechanisms. Only the first mechanism appears to be sensitive to the social gaze feedback delivered by the robot. Given that our experimental design did not include a neutral condition, we cannot conclude the direction of our manipulation on the magnitude of the SE. To estimate the direction of the SE in the two critical conditions of our protocol (mutual vs. avoiding gaze feedback), we ran a follow-up control experiment (Exp3) in which the robot was not providing any feedback to the participants.

## Experiment 3

### Materials and Methods

#### Participants

Twenty-five new participants (6 males; 1 left-handed; Mean age: 29.2 ± 11.4 years) took part in the study. All participants had normal or corrected-to-normal vision and were not informed about the purpose of the experiment. All gave their informed consent before participating and the study was conducted under the same ethical procedures and protocol as in Experiment 1. The data of five participants were excluded due to: Age of the participant above 45 years old (2 participants, see Van der Lubbe & Verleger, 2002), technical failure of the program (1 participant), because the participant committed too many errors (1 participant; error rate: 18%), or because outliers removal reduced to less than 50% the trials to be included in the analysis (1 participant). Data of twenty participants were further analyzed.

#### Apparatus, Stimuli, and Procedure

The apparatus, stimuli, and response execution were the same of Experiment 2. Stimulus presentation, response timing, and data collection were controlled as in Experiment 2.

The procedure was the same as Experiment 2, with the only exception that after a response was given, or the allowed response time elapsed, the robot did not open the eyes and did not move the neck and the eyes. At the end of each block, feedback indicating the cumulative amount of correct responses and the average response time across the task was given to participants. As in Experiment 2, instructions and feedback were given verbally by iCub. The task consisted of 3 blocks of 40 trials each. A short practice of 8 practice trials preceded the task. Since we were not interested anymore in sequential adaptations, the trial presentation order was fully randomized.

### Data Analysis

As in Experiment 2, the first trial of each block, trials that were preceded by an incorrect response, incorrect responses, and correct responses faster than 150 ms and slower than 1000 ms were excluded from the analysis (9.2% of the administered trials). Mean correct RTs for corresponding and non-corresponding trials were compared using paired samples t-tests.

### Results

The analysis on RTs showed that responses were faster for corresponding (M = 420 ms, SE = 1.7 ms) than for non-corresponding trials (M = 454 ms, SE = 1.7 ms), t(19) = 4.83, p < .001, d = 1.08.

Results of Experiment 3 showed that when participants were not provided with any feedback from the robot, our setup induced a Simon effect of 34 ms.

## The role of embodiment: Comparison between Exp1 and Exp2

**Figure 3:**
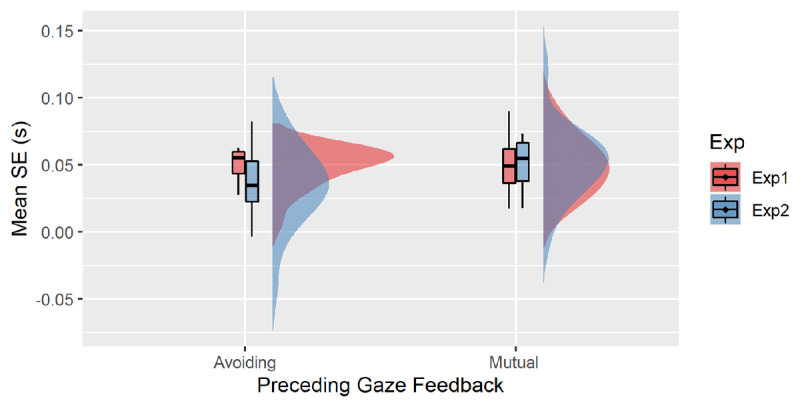
Mean Simon Effect (s) plotted as a function Preceding Gaze Feedback (Avoiding vs. Mutual) across experiments.

To assess the effect of embodiment, we conducted an additional analysis to compare the data of the two experiments. The IQR method on the average RTs of correct trials for each participant was applied to identify possible outliers. Indeed, 1 participant showed an average RT above the upper limit (Q3+1.5 IQR). Thus we ran the analysis excluding this participant on a sample size of N = 39. Mean RTs were entered into an ANOVA with Trial n Correspondence (C vs. NC) and Preceding Gaze Feedback (Avoiding vs. Mutual) as within-subject factors and Experiment (Exp1 vs. Exp2) as a between-subjects factor. In addition to the main effect of Trial n Correspondence, F(1, 37) = 209.67, p < .001, η_p_^2^ = .85, the analysis revealed a main effect of Preceding Gaze, F(1, 37) = 5.87, p = .020, η_p_^2^ = .14 and a significant two-way interaction Trial n Correspondence * Preceding Gaze Feedback, F(1, 37) = 4.90, p = .033, η_p_^2^ = .12. Importantly, the three-way interaction was significant, F(1, 37) = 5.53, p = .039, η_p_^2^ = .11. Independent T-test showed a marginally significant difference in the magnitude of the SE, t(37) = 2.0, p = .056, d = .63 following avoiding gaze feedback (see ***Figure 3***). No significant difference in the SE occurred across experiments following a Mutual gaze, t < 1. No other main effects or interactions were significant, all ps > .326.

## The effect of social gaze: Comparison between Exp2 and Exp3

We ran two exploratory analyses to compare the SE elicited following Mutual and Avoiding gaze feedback with the SE when no feedback was provided. Specifically, we compared the SE of Exp 3 with the SE of Exp2 for mutual gaze and avoiding gaze conditions separately. Mean RTs were entered into an ANOVA with Trial n Correspondence (C vs. NC) as within-subject factors and Gaze Condition (Avoiding or Mutual vs. Neutral) as a between-subjects factor.

### Avoiding vs Neutral

Apart from the main effect of Trial n Correspondence, F(1, 38) = 51.70, p < .001, η_p_^2^ = .585, no other main effects or interactions were significant, all ps > .470.

### Mutual vs Neutral

**Figure 4:**
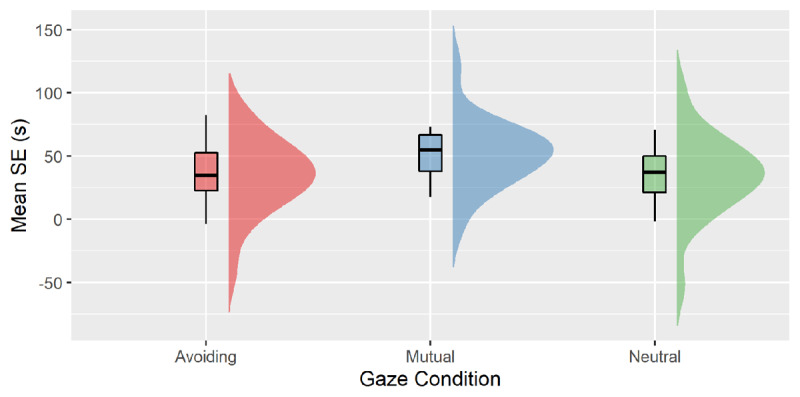
Mean Simon Effect (s) plotted as a function Gaze Condition (Avoiding vs. Mutual vs. Neutral).

The analysis showed a main effect of Trial n Correspondence, F(1, 38) = 87.22, p < .001, η_p_^2^ = .70. The two-way interaction Trial n Correspondence* Gaze Condition showed a tendency to a significant effect, F(1, 38) = 3.39, p = .073, η_p_^2^ = .08. Independent-samples t-test showed that the 34-ms SE of the Neutral gaze condition marginally differed from the 51-ms SE following mutual gaze feedback, t(38) = 1.84, p = .073, d = 0.58, see ***Figure 4***.

## General Discussion

The main goal of the present study was threefold: i) investigating whether the social gaze feedback of a robot can modulate cognitive control; ii) understanding if the social gaze feedback of a robot affects cognitive control through within-trial conflict resolution (i.e., adjusting task parameters and priority settings before the occurrence of cognitively demanding events) or it acts through trial-by-trial adaptations; iii) testing the impact of the embodied physical presence of an agent displaying social gaze. To meet these aims, in three experiments we evaluated both the SE and trial-by-trial adaptations as a function of the type of feedback participants received in the previous trial from the iCub robot, namely, mutual or avoiding gaze. Across experiments, we compared the effect of the social gaze feedback between screen-based (Exp1) and embodied setups with the robot’s physical presence (Exp2 and Exp3).

Results of Experiment 1 revealed that the 51 ms SE was modulated by the correspondence of the preceding trial. Specifically, in line with previous studies, the typical sequential effects occurred (e.g., Ciardo et al., 2019; Iani et al., 2014; 2016) with a larger SE evident following a corresponding (59 ms) than a non-corresponding trial (28 ms). Moreover, Experiment 1 showed that when the social gaze feedback was manipulated in a screen-based setup, it did not modulate either conflict resolution or conflict adaptations. In Experiment 2, apart from the expected correspondence and trial-by-trial adaptation effects, the results showed faster responses following avoiding gaze than mutual gaze feedback. Most importantly for the aim of the present study, results of Experiment 2 showed that when the social gaze feedback of the robot was manipulated in a setup involving physical embodied presence, the SE was larger in magnitude following mutual gaze feedback (51ms) than following avoiding gaze (35ms). No effect of social gaze emerged for trial-by-trial adaptations as indicated by non-significant three-way interaction. Given that Experiment 2 did not include a no-gaze condition, we ran the control Experiment 3 to estimate the baseline magnitude of the SE elicited by our embodied setup.

Our results showed that when the robot did not display social gaze feedback, a SE of 34 ms was observed, suggesting that our setup elicited overall a SE similar in magnitude to those reported in previous screen-based studies using a similar procedure (e.g. D’ascenzo et al., 2018; Xiong & Proctor, 2015). Such result is not in line with previous evidence showing that conflict effects, such as the Stroop effect, are reduced in the presence of another agent (Huguet et al.,1999; Spatola et al., 2018; see Belletier et al 2019 for a review).

Overall, our results suggest that mutual gaze feedback affects conflict resolution in the subsequent trial but only with the physical presence of the robot, i.e. when the gaze behaviour can be established in real-time.

Our results extend earlier findings showing for the first time that social gaze feedback (even when displayed by an artificial agent such as a humanoid robot) affects cognitive control. Specifically, its modulatory effect on cognitive conflict appears to be in contrast to previous studies showing that the negative affective state induced by losses or negative emotion enhances cognitive control resulting in a smaller interference effect in conflict tasks (see van Steenbergen, 2015 for a review) and do not support the affective-congruent hypothesis. Our results suggest that following mutual gaze feedback, **conflict resolution** might be reduced in efficiency. Such a result is in line with evidence showing that mutual, but not avoiding, gaze interferes with the inhibition of task-irrelevant information (Kompatsiari et al., 2021a). Specifically, in a series of recent studies, Kompatsiari and colleagues showed that mutual gaze delays attention disengagement from the non-predictive cue in a gaze cueing task. As a result, the distracting effect of gaze cues was prolonged in time even when the stimulus onset asynchrony was equal to 1s (Kompatsiari et al., 2021b). Using a decision-making task Belkaid et al., (2021) showed that mutual gaze delayed reaction times by increasing the decision threshold. Also, iCub’s mutual gaze was associated with higher alpha synchronization in the EEG signal, indicating that during mutual gaze with the robot, suppression mechanisms were activated. In a similar vein, in our study, mutual gaze may have affected attention control on response selection. As a consequence, participants were delayed in solving the response conflict elicited by the task-irrelevant spatial feature in non-corresponding trials. Interestingly, our results showed that the effect of mutual gaze occurs not only when social gaze is provided as a cue (Kompatsiari et al., 2021b) but also when the social gaze is manipulated as feedback, as its effect was transferred to the processing of a stimulus presented in a subsequent trial. Our results can be considered in the context of the proposal that social brain networks may be in antagonism with the frontoparietal attentional network (e.g. Bossi et al., 2020; Anticevic et al., 2012; Fox et al., 2005). In line with this reasoning, it could be that when presented with a socially relevant event, like the communicative mutual gaze, the recruitment of social brain areas may act against the attentional focus. Future studies should systematically address this hypothesis both at the neural and behavioural levels. One intriguing issue that remains to be addressed is whether social signals interfere at the target processing level or the response selection stage. This could be examined by presenting social gaze at the same time as the target stimulus.

Overall, our results indicate that social gaze feedbacks of a robot seem to be effective in modulating conflict resolution but not for conflict adaptions. A similar dissociation has been shown in literature in those studies that investigated individual differences in the cognitive control mechanism. For instance, Iani et al. (2014) showed that while conflict resolution in a given trial is affected by age of the participants, the trial-by-trial adaptations are not. Specifically, the authors reported a larger SE for first-grade children (6–7 years) compared to second-graders (7–8 years) children, however, the reduction in the magnitude of the SE following a conflict event (i.e. a non-corresponding trial) was comparable across groups (see also Larson, et al., 2012; for similar results in elderly).

The lack of modulation in the trial-by-trial adaptations could be due to the fact that in our study, iCub’s gaze feedback was independent of participants’ performance. Strümer and colleagues (2011) showed that, in a Simon task, monetary gains and losses in between trials affected trial-by-trial adaptations only when they were contingent on the actual performance (i.e., reward and penalties were assigned to the 25% fastest and slowest responses) but not when they occurred randomly. Similar results have been reported also using different conflict tasks, such as the Flanker task and the task-switch paradigm (Braem et al., 2012). Future studies should investigate the effect of the social gaze feedbacks of a robot in affecting conflict resolution and conflict adaptations in a response-contingent way. For instance, mutual and avoiding gaze feedbacks could be provided to reward or punish the 25% fastest and slowest responses.

Finally, by comparing the effect of the social gaze feedback between screen-based and embodied setups, our results show that only the latter was effective in affecting conflict resolution. Such a result extends previous evidence showing that when investigating social gaze, screen-based protocols might lack ecological validity (Kompatsiari et al., 2021b). For instance, Hietanen et al., (2008) showed that real-time mutual gaze was associated with greater sympathetic arousal (skin conductance responses), than averted gaze. Similarly, Pönkänen et al. (2011) reported different EEG asymmetries for faces that were presented live through an electronic shutter and those that were presented as pictures on a computer screen. It has been proposed that real-time social gaze is likely to play a greater role in influencing sensations of intimacy, experienced self-relevance, and awareness about being seen by another person (or a robot) who is physically present, as compared to seeing a picture of a face on a computer screen (Hietanen et al., 2008, Pönkänen et al., 2011; Kompatsiari et al., 2018; 2019).

We believe that our results are crucial not only for fundamental research but also for applied scenarios. The findings open up the possibility of using robots to train complex cognitive skills like self–regulation. The main advantage of embedding robots within cognitive training protocols is the possibility of maintaining good experimental control on the one hand and increased ecological validity on the other, an aspect crucial for certain mechanisms of cognition, as our data show. Through their social presence, robots can interact in real-time with the user in a shared environment. Through the implementation of motivational and social behaviours, we believe that robots can improve learning. To date, robots have been mostly used in rehabilitation protocols for motor and social skills, such as joint attention (see Chevalier et al., 2019 for a review). However, taking into consideration that cognitive control, and response inhibition specifically, is impaired not only in aging but also in several psychiatric disorders, such as but not limited to, eating and mood disorders, Tourettes’ syndrome, and obsessive-compulsive spectrum disorders, we propose that also cognitive control training protocols can benefit from the implementation of robots. For instance, several studies showed that children with attention-deficit/hyperactivity disorder (ADHD) report larger Simon and Flanker effects when compared to children with typical development (for a review see Mullane, Corkum, Klein, & McLaughlin, 2009). Also, it has been shown that conflict resolution and conflict adaptation mechanisms are differently impaired in patients with eating disorders (Bartholdy et al., 2017). Using a stop-signal task Bartholdy and colleagues showed that conflict resolution was enhanced in women with anorexia compared to healthy controls, whereas no difference across groups was reported for reactive control mechanisms. Similarly, Sellaro and Colzato (2017) reported a larger SE for overweight individuals compared to normal-weight controls.

To conclude, the present study shows that robots’ social gaze feedback modulates conflict resolution in a Simon task but not conflict adaptations. The modulatory effect was observed for the embodied setup in which the robot could engage or avoid eye contact in real-time, opening up the way to a new potential application of robots in protocols aiming to investigate, and train, cognitive control and self-regulation in both healthy and clinical populations.

## Data Accessibility Statement

Data, videos, and stimuli are available at *https://osf.io/nvcz6/*.

## Footnotes

Please note that when talking about affective states we refer to positive or negative states that could be driven both by motivational factors and/or emotions.
